# A Flexible Yet Robust 3D-Hybrid Gel Solid-State Electrolyte Based on Metal–Organic Frameworks for Rechargeable Lithium Metal Batteries

**DOI:** 10.3390/gels10120812

**Published:** 2024-12-10

**Authors:** Ruliang Liu, Jiaqi Xue, Lijun Xie, Huirong Chen, Zhaoxia Deng, Wei Yin

**Affiliations:** School of Chemistry and Materials Science, Guangdong University of Education, Guangzhou 510303, China; 17652806905@163.com (J.X.); chen-huirong@outlook.com (H.C.); dengzhaoxia@gdei.edu.cn (Z.D.); yinwei@gdei.edu.cn (W.Y.)

**Keywords:** solid electrolytes, gel, Li metal batteries, MOFs, composite

## Abstract

Compared to traditional liquid electrolytes, solid electrolytes have received widespread attention due to their higher safety. In this work, a vinyl functionalized metal–organic framework porous material (MIL-101(Cr)-NH-Met, noted as MCN-M) is synthesized by postsynthetic modification. A novel three-dimensional hybrid gel composite solid electrolyte (GCSE-P/MCN-M) is successfully prepared via in situ gel reaction of a mixture containing multifunctional hybrid crosslinker (MCN-M), lithium bis(trifluoromethanesulfonyl)imide (LiTFSI), ethylene carbonate (EC), diethylene glycol monomethyl ether methacrylate (EGM) and polyethylene (vinylidene fluoridee) (PVDF). Benefiting from the excellent mechanical properties, rich pore structure, and numerous unsaturated metal sites of GCSE-P/MCN-M, our GCSE-P/MCN-M exhibits excellent mechanical modulus (953 MPa), good ionic conductivity (9.3 × 10^−4^ S cm^−1^) and wide electrochemical window (4.8 V). In addition, Li/LiFePO_4_ batteries based on GCSE-P/MCN-M have also demonstrated excellent cycling performance (a high-capacity retention of 87% after 200 cycles at 0.5 C). This work provides a promising approach for developing gel solid-state electrolytes with high ion conduction and excellent safety performance.

## 1. Introduction

With the increasing demand for long endurance in electric vehicles, developing batteries with high energy density and safety is also crucial for the electric vehicle market. The energy density of lithium metal batteries (LMBs) is 2–6 times that of traditional lithium-ion batteries, which has attracted widespread attentions [1,2,3,4]. For LMBs, lithium metal has an ultra-high theoretical specific capacity (3860 mAh g^−1^) and an extremely low potential (3.04 V vs. standard hydrogen electrode) and is considered as one of most promising anode materials [5,6,7,8]. When employing traditional liquid electrolytes, the thermodynamic properties of lithium metal are unstable. During the process of lithium stripping/plating, the growth of lithium dendrites may puncture the separator, causing battery short circuits and triggering battery heating and explosion. These potential safety issues limit the application of lithium metal anodes [9,10]. Solid state electrolytes with high mechanical/thermal stability and good electrolyte/electrode compatibility are considered effective means of solving lithium dendrite-related problems [11,12]. Therefore, from the perspective of balancing high energy density and intrinsic safety, it is necessary to further design and develop high-performance solid electrolytes that are compatible with lithium metal anodes.

Solid state polymer electrolytes (SPEs) have been widely used in lithium metal batteries due to their advantages such as light weight, good flexibility, and easy processing [13]. However, low room temperature ionic conductivity, high electrochemical interface impedance, and low ion migration number remain significant challenges for the development of SPEs [14,15]. Since Weston and Steele first reported composite electrolytes with Al_2_O_3_ fillers [16], the introduction of inorganic nanomaterials into polymer matrices to combine the advantages of inorganic and organic electrolytes and construct new composite solid electrolytes (CSEs) has attracted the attention of many researchers. It is widely believed that ceramic nanoparticles can promote local structural modification of polymer matrices, leading to an increase in the concentration of free lithium ions, which can quickly move throughout the entire conductive path of the ceramic extension surface, improving ion conductivity [17,18,19]. Moreover, introducing inorganic nanoparticles is beneficial for improving the thermodynamic and mechanical properties of polymer electrolytes [20,21,22,23]. Therefore, designing the composition, structure, and film-forming process of fillers with polymers has become a hot research topic.

Metal–organic frameworks (MOFs) have been widely used in various fields such as catalysis, separation and purification, sensors, and gas storage [24,25]. CSE constructed with MOFs as fillers also exhibits good ionic conductivity and interfacial properties [26,27,28]. Liu et al. [29] first synthesized a novel MOF (MOF-5) and prepared CSE by combining it with PEO/LiTFSI system. They found that when the material composition was PEO/LiTFSI (EO:Li^+^ = 10)/MOF-5 (10 wt%), the ionic conductivity of the electrolyte material reached 3.16 × 10^−6^ S cm^−1^ at 25 °C. Recently, a great number of efforts have been devoted to enhancing the electrochemical performance MOF-based CSE. For example, Mai et al. [30] prepared a composite polymer electrolyte with fast ion transport and good mechanical support through layered self-assembly of metal–organic frameworks (MOFs). Due to a large number of Lewis sites, a continuous ion transport network, and a robust polyimide framework, the resulting CSE provides high ion conductivity of 4.08 × 10^−4^ S cm^−1^ at 30 °C and effectively improves the cycling stability of lithium metal batteries. Subsequently, He et al. [31] report on a composite solid electrolyte (h-PAN@MOF) coupling polyacrylonitrile fiber network and interconnected metal–organic framework coating. h-PAN@MOF has an efficient 3D channel for Li^+^ transport, exhibiting high ion conductivity of 1.03 × 10^−3^ S cm^−1^ and high tensile strength (20.84 MPa). Finally, the full battery-based h-PAN@MOF achieved an excellent cycle of 1000 cycles at 5C. However, due to the lack of suitable functional design for MOFs, the interface compatibility of various components in MOF-based composite solid electrolytes is poor, which leads to difficulties in ion transport inside the composite membrane, high impedance between the composite membrane and the electrolyte interface, and poor mechanical properties of the composite membrane. Designing and preparing functional MOF materials to construct high-performance composite solid electrolytes remains a huge challenge.

In this work, we first synthesized MOF nanoparticles (MIL-101(Cr)-NH_2_, noted as MCN) and used a postsynthetic modification strategy to obtain ethylene functionalized MOFs (MIL-101(Cr)-NH-Met, noted as MCN-M). Then, a novel three-dimensional (3D) hybrid gel composite solid electrolyte (GCSE-P/MCN-M) was successfully prepared by simple scratch coating and in situ gel formation. This kind of 3D GCSE-P/MCN-M has the following characteristics. Firstly, MCN-M can provide super cross-linking sites, which can significantly improve the mechanical properties and thermal stability of the electrolyte membrane. Secondly, the rich microporous structure, unsaturated metal sites and abundant ethoxy groups are beneficial for accelerating ion transport and confining the movement of anions. Finally, a large number of hydrogen bonds between polymers and MCN-M can effectively improve the interfacial compatibility between the components of composite electrolyte and between electrolyte and electrode. Therefore, this type of GCSE is applied to LMBs, exhibiting excellent compatibility between electrolyte and lithium metal electrode. In addition, Li/LiFePO_4_ batteries based on GCSE-P/MCN-M have also demonstrated excellent cycling performance (200 cycles at 0.5 C, with an average specific capacity of up to 124.7 mAh g^−1^).

## 2. Results and Discussion

**Figure 1:**
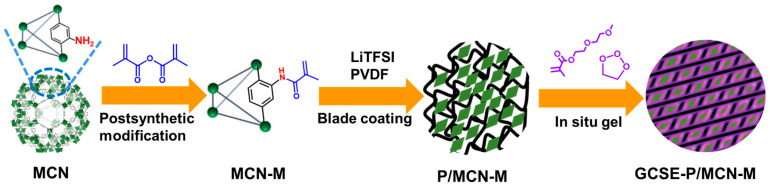
Preparation of GCSE-P/MCM-M.

The preparation process of GCSE-P/MCN-M membrane is schematically depicted in Figure 1. MIL-101(Cr)-NH_2_ (MCN) was first synthesized and functionalized with methacrylamide groups to obtain MIL-101(Cr)-NH_2_-Met (MCN-M) by a postsynthetic modification strategy. Second, the suspension containing MCN-M, lithium bis(trifluoromethanesulfonyl)imide (LiTFSI) and polyvinylidene fluoride (PVDF) was scraped onto a Teflon plate to obtain the P/MCN-M membrane. GCSE-P/MCN-M was finally prepared by dripping a mixture of diethylene glycol monomethyl ether methacrylate (EGM), ethylene carbonate (EC) and thermal initiation agents into P/MCN-M membrane and subsequently irradiated under heating treatment. The MOF/polymer hybrid membrane (GCSE-P/MCN-M) prepared using this method can effectively improve the aggregation of MOF nanoparticles and the poor compatibility between organic and inorganic components.

**Figure 2:**
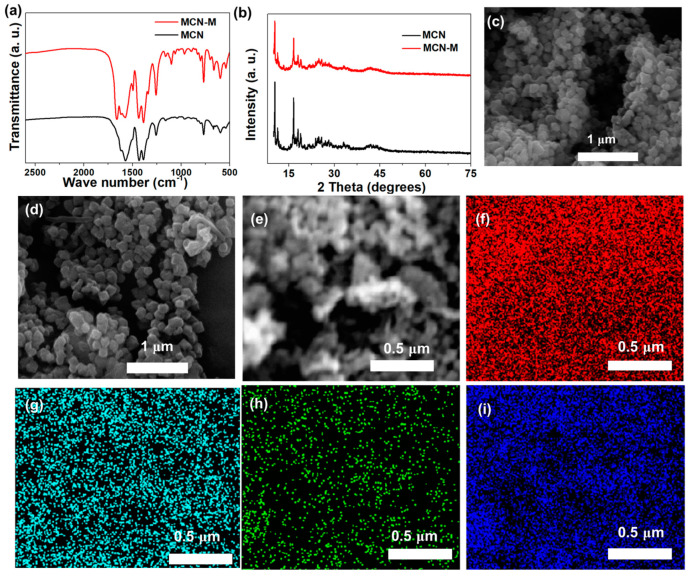
(**a**) FTIR spectra and (**b**) PXRD patterns of MCN and MCN-M. SEM images of (**c**) MCN and (**d**) MCN-M. SEM image of (**e**) MCN-M and corresponding elemental mapping images of (**f**) C, (**g**) Cr, (**h**) N and (**i**) O.

As shown in Figure 2a, the Fourier Transform Infrared (FTIR) spectrum of MIL-101(Cr)-NH_2_ shows three characteristic peaks at 1058, 1389 and 1568 cm^−1^, which are attributed to a stretching vibration of Cr-O, the tensile vibration absorption of C=C and C=O in terephthalic acid, indicating that MCN was successfully prepared [32,33]. The FTIR spectrum of MCN-M demonstrates a peak at 1631 cm^−1^, attributed to the stretching vibration peak of C=C. Meanwhile, a new characteristic peak appeared at 1665 cm^−1^, attributed to the C=O band in the amide group of methacrylamide [34]. The IR results confirm that methacrylamide groups are successfully grafted onto MCN by the postsynthetic modification method. A powder X-ray diffraction (PXRD) test is also conducted. As displayed in Figure 2b, MCN exhibits obvious characteristic diffraction peaks at 10°, 12.5°, 17°, 18° and 19°, which are consistent with the standard diffraction peaks described in the literature [32,33], further demonstrating our successful preparation of MCN. Compared with MCN, the PXRD spectrum of MCN-M does not show significant changes, indicating that this postsynthetic modification method does not damage the integrity of the MCN crystal structure. The scanning electron microscopy (SEM) images of MCN and MCN-M show similar octahedral nanostructure morphology (Figure 2c,d), and the size of the nanoparticle is around 100 nm, further confirming that the postsynthetic modification strategy can effectively inherit the microstructure of the pristine MOFs. Moreover, the elemental mapping images (Figure 2e–i) indicate that the C, Cr, N and O elements are evenly distributed in the MCN-M nanoparticle, further confirming that the postsynthetic modification strategy does not damage the micro nano structure of the MCN. The N_2_ adsorption–desorption properties of the pure MCN and MCN-M are also measured. As shown in Figure S1, the Brunauer–Emmett–Teller (BET) surface area of MCN and MCN-M are calibrated to be as high as 1501 and 1132 m^2^ g^−1^, respectively. In addition, according to the DFT (Density Function Theory) pore size distribution curve, the micropore width of MCN and MCN-M is measured to be around 1.2 nm, indicating that the pores of MCN-M are not blocked by modifying functional groups.

**Figure 3:**
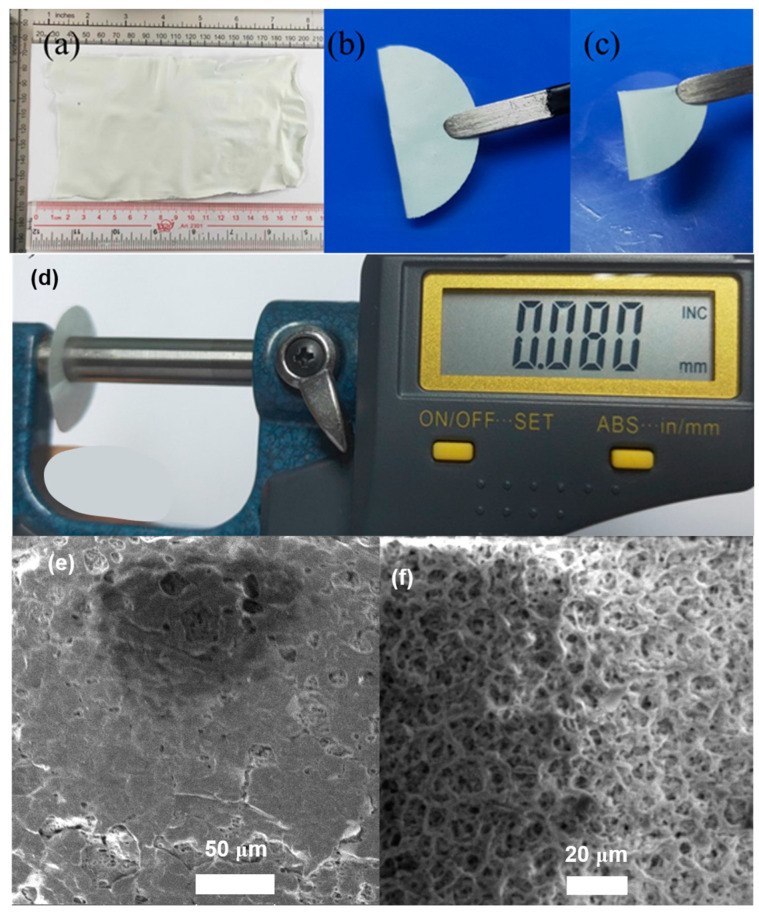
(**a**) Digital photo of a large piece of P/MCN-M membrane. Digital photos of (**b**) bending and (**c**) folding of P/MCN-M membrane. (**d**) Digital photo of thickness measurement of P/MCN-M membrane. SEM images of (**e**) PVDF membrane and (**f**) P/MCN-M membrane.

We homogeneously mixed quantitative MCN-M as a multifunctional filler with PVDF and LiTFSI via ultrasound and stirring treatment, and then used a simple and efficient scraping process to prepare large-area porous organic–inorganic composite membrane P/MCN-M (Figure 3a). As shown in Figure 3b,c, the P/MCN-M composite membrane can be bent and folded freely, exhibiting good flexibility. The thickness of the P/MCN-M composite membrane is as low as 80 μm (Figure 3d). Compared with pure PVDF-based polymer membrane, the composite membrane with MCN-M multifunctional filler added has a richer pore structure, with a surface porosity of about 12.1% (Figure 3e,f). This 3D porous nano network structure can significantly improve the liquid absorption rate of the membrane and facilitate the rapid transport of lithium ions. Gel composite solid electrolyte (GCSE-P/MCN-M) can be finally obtained by in situ gel reaction of composite membrane adsorbed with EC and EMG. The mechanical properties of solid electrolytes are very important for the safety of batteries. The force–depth curves are displayed in Figure S2, which shows that the elastic moduli of GCSE-P/MCN-M are as high as 953 MPa, which is much better than pure PVDF-based gel solid electrolyte (GSE-P) (217 MPa). The results indicate that MCN-M mutifunctional filler can greatly improve the mechanical properties of conventional GSE. This excellent mechanical performance of GCSE-P/MCN-M is beneficial for suppressing the uncontrolled growth of lithium dendrites during the cycling process of lithium metal batteries.

**Figure 4:**
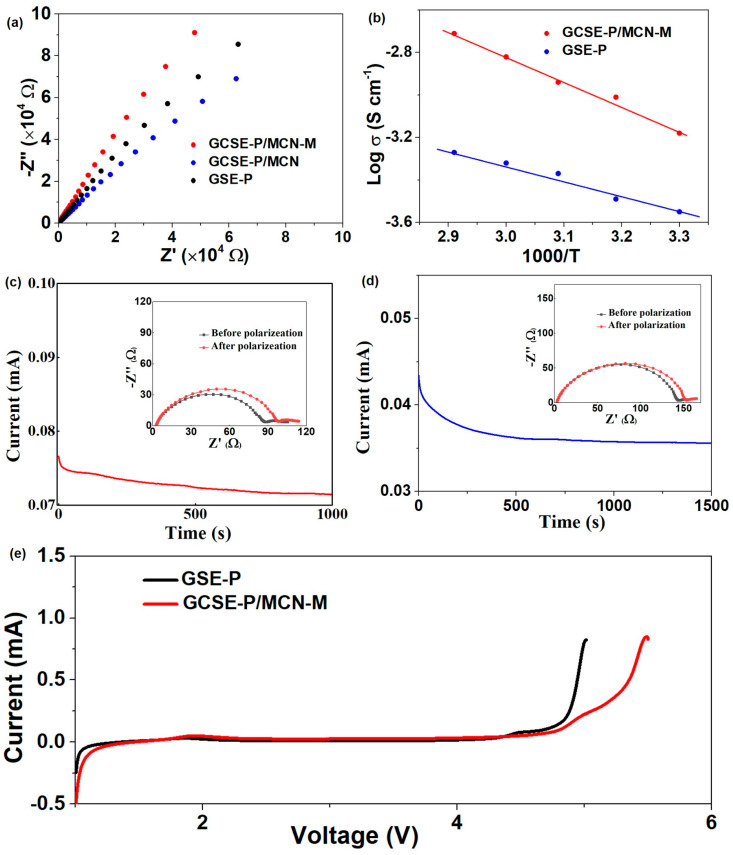
(**a**) Nyquist plots of stainless-steel symmetrical cells with GSE-P, GCSE-P/MCN and GCSE-P/MCN-M. (**b**) The ionic conductivities of GSE-P and GCSE-P/MCN-M membrane as a function of temperature. Chronoamperometry profiles of Li/Li symmetric cells with (**c**) GSE-P and (**d**) GCSE-P/MCN-M at 10 mV of polarization (inset: EIS curves before and after polarization). (**e**) LSV curves of GSE-P and GCSE-P/MCN-M at a scan rate of 2 mV s^−1^.

Thermal stability is an important characteristic for GSE. In order to investigate the thermal stability of different membranes, the heat resistance of GCSE-P/MCN-M, GSE-P and PP membranes through high-temperature heat treatment is tested. The macroscopic size changes of membranes at different temperatures are shown in Figure S3. Commercial PP membrane began to show significant size changes at 100 °C and fully contracted at 150 °C. GSE-P membrane shrinks at 150 °C. In contrast, the macroscopic morphology of GCSE-P/MCN-M membrane remains unchanged, and no significant shrinkage can be observed during the entire heating process (25–180 °C). These results indicate that GCSE-P/MCN-M have good thermostability in the macroscopical morphology. Ion conductivity of different solid electrolytes is evaluated according to the EIS test at 25 °C (Figure 4a). The ion conductivity of GCSE-P/MCN-M is as high as 9.3 × 10^−4^ S cm^−1^, which is significantly better than GCSE-P/MCN (5.3 × 10^−4^ S cm^−1^) and GSE-P (2.6 × 10^−4^ S cm^−1^. This indicates that both MCN-M materials can improve the ionic conductivity of electrolytes. The impedance spectrum shows that the relationship between the ion conductivity and temperature of GSE-P/MCN-M and GSE-P at 30–70 °C follows a typical Arrhenius linear equation, and GCSE-P/MCN-M has more efficient ion transport characteristics, which can promote the rapid diffusion of lithium ions (Figure 4b and Figure S4). The lithium-ion transference number (*t*_Li+_) can reflect the contribution of lithium ions in the transport current. A high *t*_Li+_ can effectively reduce electrode polarization and improve charge discharge efficiency. The *t*_Li+_ tests for a series of GSE are conducted (Figure 4c,d). The *t*_Li+_ of GCSE-P/MCN-M was 0.67, which was superior to GSE-P (0.26). The result indicates that MCN-M and PEGM can construct rich and efficient lithium-ion transport pathways, which are beneficial for suppressing concentration gradient formation, lithium dendrite growth, and interface resistance increase [34,35]. In addition, GCSE-P/MCN-M has a better *t*_Li+_ than some reported quasi-solid electrolytes, such as FEC-SPE [36] (*t*_Li+_ is 0.57), SLE [37] (*t*_Li+_ is 0.51), and SiO_2_-GPE [38] (*t*_Li+_ is 0.45). Furthermore, as displayed in Figure 4e, the Li/SS battery based on GCSE-P/MCN-M has a wider electrochemical working window (~4.8 V), significantly better than GSE-P (~4.3 V), which is beneficial for stabilizing the electrode electrolyte interface and achieving stable operation of lithium metal batteries.

**Figure 5:**
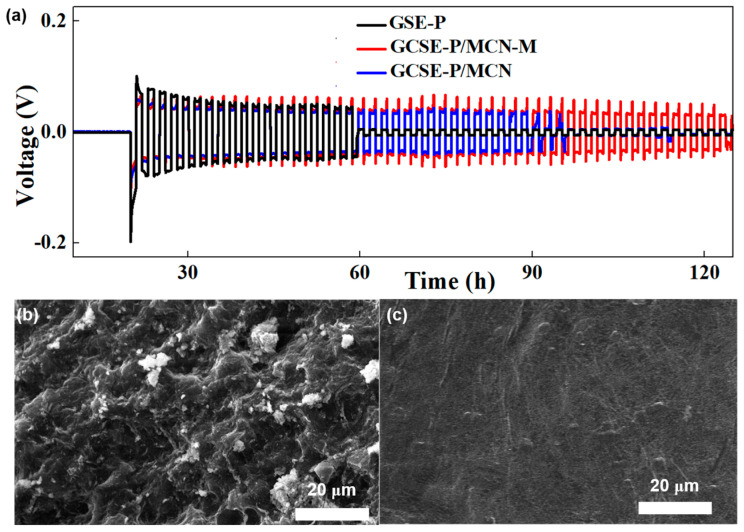
(**a**) Voltage–time profiles of Li/Li symmetric cells using GSE-P, GCSE-P/MCN and GCSE-P/MCN-M at 1 mA cm^−2^ with a capacity of 1 mAh cm^−2^. SEM images of lithium anode surface from cycled Li/Li symmetric cells with (**b**) GSE-P and (**c**) GCSE-P/MCN-M.

To further investigate the stability of the interface between the Li metal and GCSE-P/MCN-M during Li plating and stripping, Li metal symmetric cells with three kinds of GCSE are assembled. As displayed in Figure 5a, the Li|GCSE-P/MCN-M|Li cell can cycle stably for over 120 h without any short circuit at 1 mA cm^2^ with a capacity of 1 mAh cm^2^. In sharp contrast, the Li|GCSE-P/MCN|Li cell exhibits irregular voltage fluctuations after only 87 h of cycling. When assembling Li symmetric cells with GCSE-P, the Li|GSE-P|Li cell shows an obvious short circuit after only 60 h of cycling. The results indicate that GCSE-P/MCN-M is beneficial for uniform Li deposition/dissolution and improves the cycling stability of lithium metal anode. Moreover, to investigate the inhibitory effect of GCSE-P/MCN-M on lithium dendrite growth, surface morphology of fresh Li metal and Li metal from Li/Li symmetric batteries after cycling is observed by SEM. As shown in Figure 5b, the surface lithium metal from the Li|GSE-P|Li cell after cycling shows many uneven pores, indicating that lithium metal is severely corroded during lithium deposition/stripping. This phenomenon may be attributed to uncontrolled growth of lithium dendrites caused by uneven lithium deposition, which further results in the rupture of the native solid electrolyte interphase film and the generation of “dead lithium”. It is worth noting that after the same cycles, the Li surface of the Li|GCSE-P/MCN-M|Li cell appears relatively flat and dense, and no obvious lithium dendrites are observed (Figure 5c). These results demonstrate that the 3D network skeleton formed by the super crosslinking between rigid MCN-M fillers and PEGM provides high mechanical strength, which can effectively suppress the growth of lithium dendrites. In addition, the synergistic effect of abundant ethoxy groups of polymers and rich micropore structures can regulate and optimize the lithium-ion transport behavior, which helps promote the uniform deposition of lithium metal.

**Figure 6:**
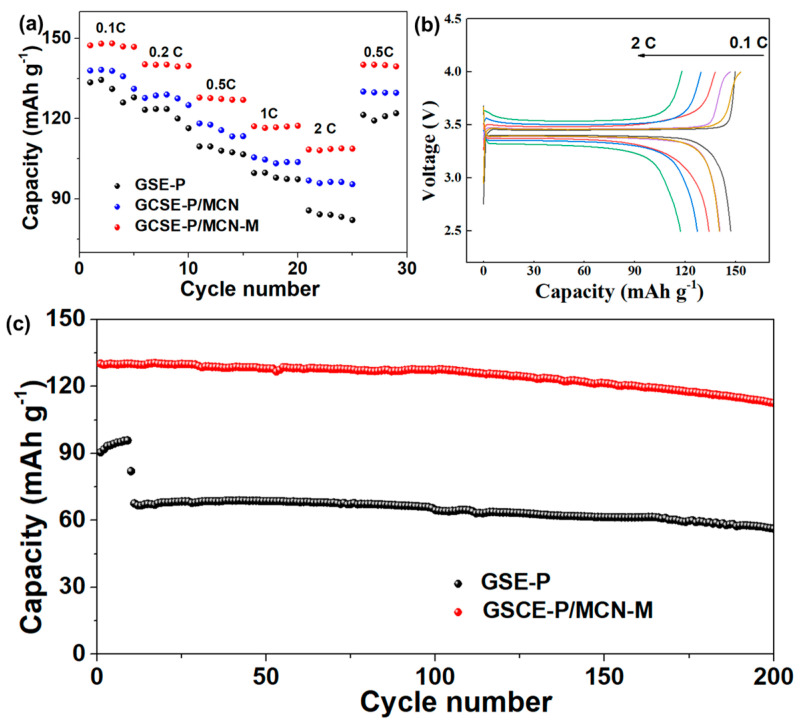
(**a**) Rate capability of Li/LiFePO_4_ cells with GSE-P, GCSE-P/MCN and GCSE-P/MCN-M at rates from 0.1 to 2 C. (**b**) Charge/discharge profiles of Li/LiFePO_4_ cell with GCSE-P/MCN-M at various rates. (**c**) Long-term cycling stabilities of Li/LiFePO_4_ cells with GSE-P and GCSE-P/MCN-M at a current density of 0.5 C.

In order to investigate the practicality of GCSE-P/MCN-M, Li/LiFePO_4_ (LFP) full cells based on various gel solid electrolytes are assembled and corresponding electrochemical performance tests are conducted. As shown in Figure 6a, the Li|GCSE-P/MCN-M|LFP cell exhibits excellent rate performance, providing discharge specific capacities of 141, 134, 126, 117 and 109 mAh g^−1^ at current densities of 0.1, 0.2, 0.5, 1 and 2 C, respectively, much higher than Li|GCSE-P/MCN|LFP (137.9, 129, 115.8, 103.4 and 96.3 mAh g^−1^) and Li|GSE-P|LFP (131.2, 123.6, 108, 98 and 84 mAh g^−1^) cells. When the current density returns to 0.2 C, the discharge specific capacity of the Li|GCSE-P/MCN-M| LFP battery can be maintained at 139 mAh g^−1^. As shown in Figure 6b, the charge–discharge curves of Li/LFP cells assembled using GCSE-P/MCN-M at different rates of 0.2–2 C all exhibit a clear voltage plateau and good electrochemical stability. In addition, the charge and discharge curves of the Li|GCSE-P/MCN-M|LFP cell under different cycles show a much lower polarization voltage with the increase in current density. In addition, we also test the cycling performance of the Li/LiFePO_4_ cell with different gel solid electrolytes. As shown in Figure 6c, initial specific discharge capacity of the Li|GCSE-P/MCN-M|LFP cell can reach up to 130.2 mAh g^−1^ and its discharge capacity still maintains 112.6 mA h g^−1^ with a retention of 87% after 200 cycles under a cycling current of 0.5 C. In sharp contrast, the initial capacity of the Li/LiFePO_4_ cell with GSE-P is only 90.5 mAh g^−1^, and the Li|GSE-P|LFP cell shows relatively poor cycling performance, with a capacity retention rate as low as 62% after 200 cycles at 0.5 C. The results demonstrate that GCSE-P/MCN-M can significantly enhance the cycling stability of the Li metal batteries.

## 3. Conclusions

In summary, we develop a 3D gel composite solid electrolyte (GCSE-P/MCN-M) with the incorporation of ethylene modified MOFs (MCN-M) as hyper crosslinked fillers. The GCSE-P/MCN-M can not only suppress the growth of lithium dendrites via robust 3D hyper crosslinked nano network and regulation of Li^+^ transference by MCN-M, but also enhance the interfacial compatibility between electrodes and electrolytes by means of an in situ polymerization method. Taking advantage of the multiscale interaction and in situ polymerization feature of MCN-M in the PVDF matrix, the GCSE-P/MCN-M with unique 3D cross-linked hybrid polymer network structure can significantly improve the mechanical properties and ionic conductivity. It enables the high mechanical properties (953 MPa), ultrathin thickness (80 μm), good room temperature lithium-ion conduction (9.3 × 10^−4^ S cm^−1^), and high lithium-ion transference number (0.67). As a result, Li/LFePO_4_ LBM cells based on GCSE-P/MCN-M deliver a high-capacity retention of 87% after 200 cycles at 0.5 C. These results indicate that GCSE-P/MCN-M has great practical application potential in next-generation LMBs with high energy density and safety.

## 4. Materials and Methods

### 4.1. Materials

2-Aminoterephthalic Acid (H_2_BDC-NH_2_, Aladdin, Shanghai, China), Chromium(III) nitrate nonahydrate (Cr(NO_3_)_3_·9H_2_O, Aladdin, Shanghai, China), N, N-dimethylformamide (DMF, Aladdin, Shanghai, China), polyethylene (vinylidene fluoridee) (PVDF, Mn = 534,000, Sigma, Shanghai, China), methacrylic anhydride (J&K Scientific, Beijing, China), diethylene glycol monomethyl ether methacrylate (EGM, Aladdin, Shanghai, China), lithium bis(trifluoromethanesulfonyl)imide (LiTFSI, Aladdin, Shanghai, China), ethylene carbonate (EC, Aladdin, Shanghai, China), 1- methyl-2-pyrrolidinone (NMP, Aladdin, Shanghai, China), N,N-dimethylformamide (DMF, Aladdin, Shanghai, China),Super P (Timcal, Kelude, Dongguan, China), LiFePO_4_ (LFP, Kelude, Dongguan, China), Lihium foil (99.95%, Kelude, Dongguan, China).

### 4.2. Preparation of MOFs and Gel Composite Solid Electrolyte

MIL-101(Cr)-NH_2_ (MCN) [39]: H_2_BDC-NH_2_ (0.92 g) and Cr(NO_3_)_3_·9H_2_O (2.0 g) were added to water (28 mL). The resulting mixture was stirred for 2 h at room temperature then heated at 130 °C for 24 h in a Teflon-lined autoclave. After cooling to room temperature, the solid product was isolated from the filtrate by centrifugation at 10,000 rpm and washed five times with hot ethanol. Finally, a green powder was placed in a vacuum drying oven and dried at 150 °C for 12 h.

MIL-101(Cr)-NH_2_-Met (MCN-M): The MCN was added in CH_2_Cl_2_ for ultrasonic dispersion, and 2 mL of acid anhydride was added into the mixture under nitrogen protection. The resulting mixture was kept in a closed vial for 4 days. The solids were washed several times with fresh CH_2_Cl_2_ and then dried under a vacuum at 40 °C for 12 h.

Gel composite solid electrolyte (GCSE-P/MCN-M): PVDF (2 g) was dissolved in DMF and stirred at 80 °C for 2 h to form a casting solution. A total of 0.4 g of MCN-M, 0.3 g of lithium salt and 0.1 g of SN were added to the PVDF solution, and a uniform green colloidal solution was obtained by stirring the suspension at room temperature for 2 h. The composite membrane (P/MCN-M) was formed by coating green colloidal solution on Al foil via automatic coating, and dried at 45 °C for 8 h under vacuum. The precursor solution containing EGM (40 μL), AIBN (10 mg) and EC (40 μL) was uniformly dropped onto the composite membrane. GCSE-P/MCN-M was finally obtained by heating the P/MCN-M at 65 °C for 1 h. The method of preparing GCSE-P/MCN and GCSE-P is similar to that of GCSE-P/MCN-M. For GCSE-P/MCN, MCN is used instead of MCN-M. For GSE-P, no MOF fillers were added.

### 4.3. Characterization

Fourier transform infrared spectrometry (FTIR, Brukertensor27, Bremen, Germany) in the range 4000–400 cm^−1^ was conducted using a Nicolet IS50 infrared analyzer. The morphology and microstructure of the samples were investigated by field-emission scanning electron microscopy (SEM, TESCAN MIRA LMU, Brno, Czech Republic). Elasticity moduli of membranes were measured using a dynamic ultra-micro hardness tester (DUH-W201S, Shimadzu, Kyoto, Japan) with 0.15 mN preset test force. The PXRD measurements of the samples were also carried out on X-ray diffraction (XRD, Bruker, Billerica, MA, USA) and the radiation source was Cu-Ka rays.

### 4.4. Electrochemical Measurements

The electrochemical impedance spectroscopy (EIS) was measured on the electrochemical workstation (Coster CS350, Wuhan, China) under a frequency range from 0.1 to 10^6^ Hz. The ionic conductivity (σ) of solid electrolyte membranes was calculated according to Equation (1):

(1) $$\sigma =\frac{L}{SR}$$

where *L* represents the thickness of the membrane; *S* represents the area of the electrode; and *R* represents the bulk resistance.

The lithium-ion transference number ($t_{{Li}^{+}}$) was calculated according to Equation (2):

(2) $$t_{{Li}^{+}}=\frac{I_{s}(\Delta V-I_{0}R_{0})}{I_{0}(\Delta V-I_{s}R_{s})}$$

where ΔV is the polarization voltage; $I_{0}$ and $R_{0}$ are the initial-state current and initial-state resistance, respectively; $I_{s}$ and $R_{s}$ are the steady-state current and steady-state resistance, respectively.

The LiFePO_4_ (LFP) cathode was prepared by coating the slurry of LFP powders/Super P/PVDF binder (8:1:1 by weight) on Al foil and drying at 65 °C for 15 h, which was then punched into disks with a diameter of 12 mm. CR2032 coin-type cells using LFP cathode and Li foil were assembled in an argon-filled glovebox (H_2_O < 0.1 ppm, O_2_ < 5 ppm) (MIKROUNA, Shanghai, China). The galvanostatic charge–discharge test was conducted using the NEWARE Battery Test System (Shenzhen Neware Electronics Co., Ltd., Shenzhen, China) at a voltage range of 2.5–4 V (vs. Li/Li^+^). Linear sweep voltammetry (LSV) was measured on the electrochemical workstation (Coster CS350, Wuhan, China) using the cell configuration of Li/stainless-steel at a scan rate of 2 mV s^−1^.

## Data Availability

The original contributions presented in this study are included in the article/Supplementary Materials. Further inquiries can be directed to the corresponding authors.

## Supplementary Materials

The following supporting information can be downloaded at: https://www.mdpi.com/article/10.3390/gels10120812/s1. Figure S1. (a) N_2_ adsorption isotherms of MCN and MCN-M at 77 K. (b) Pore size distributions of MCN and MCN-M; Figure S2. Force-depth curves of GSE-P and GCSE-P/MCN-M membranes under the same maximum force (0.15 mN); Figure S3. Digital photos of thermal shrinkage for (a) PP membrane, (b) PVDF membrane and (c) P/MCN-M membrane after heating at different temperatures; Figure S4. Nyquist plots of stainless-steel symmetrical cells with (a) GSE-P and (b) GCSE-P/MCN-M at various temperatures from 303 to 343 K.
