# Supplementary material for: A Flexible Yet Robust 3D-Hybrid Gel Solid-State Electrolyte Based on Metal–Organic Frameworks for Rechargeable Lithium Metal Batteries
Source: Gels. 2024 Dec 10;10(12):812. doi: 10.3390/gels10120812 (PMC11675897; doi:10.3390/gels10120812)
Supplement: Supplementary file 1 [file gels-10-00812-s001.zip › gels-3329037-supplementary.pdf]

Supporting Information

A Flexible yet Robust 3D-Hybrid Gel Solid-state Electrolytes based on Metal-organic Frameworks for Rechargeable Lithium Metal Batteries

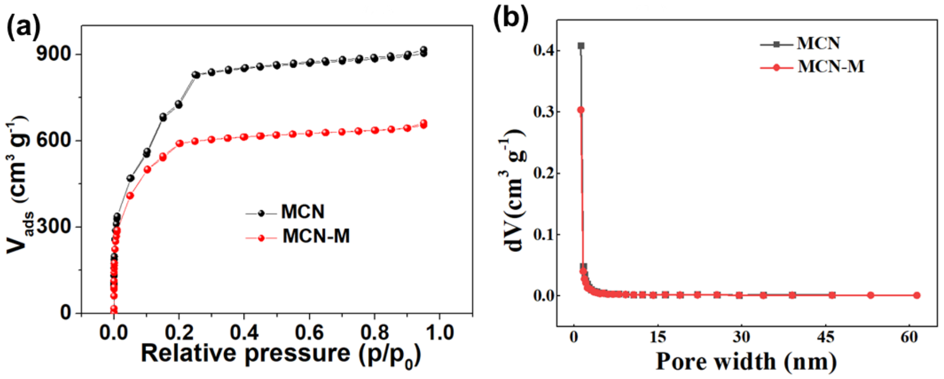

Figure S1. (a) N_2 adsorption isotherms of MCN and MCN-M at 77 K. (b) Pore size distributions of MCN and MCN-M.

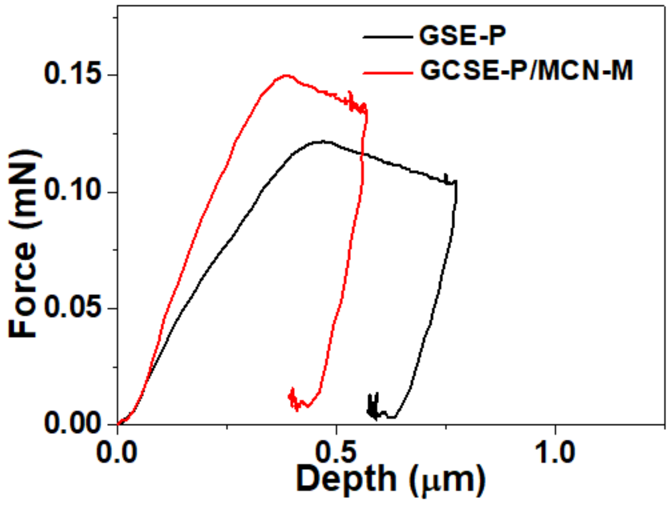

Figure S2. Force-depth curves of GSE-P and GCSE-P/MCN-M membranes under the same maximum force (0.15 mN).

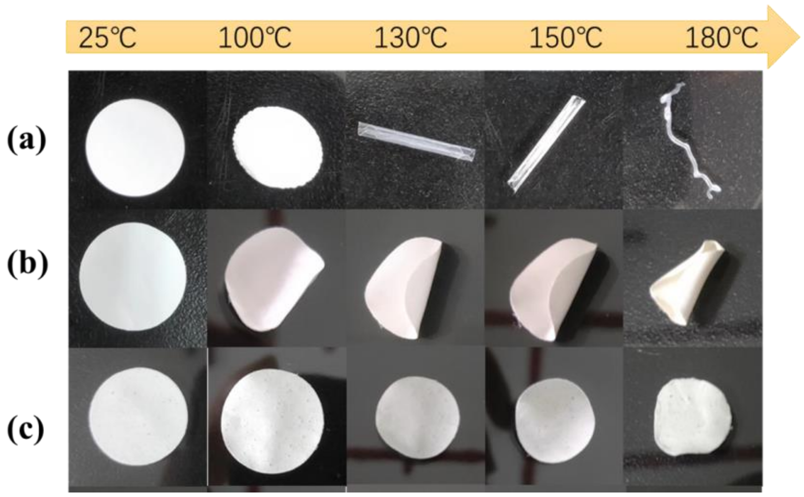

Figure S3. Digital photos of thermal shrinkage for (a) PP membrane, (b) PVDF membrane and (c) P/MCN-M membrane after heating at different temperatures.

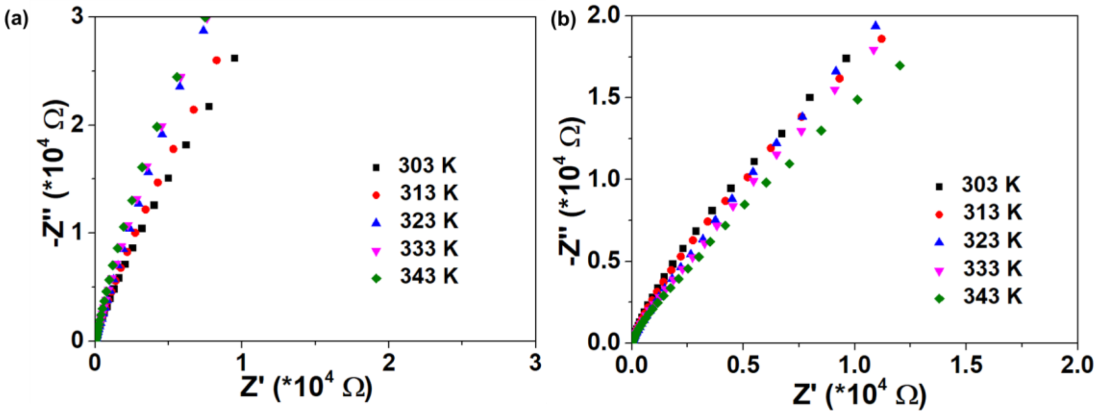

Figure S4. Nyquist plots of of stainless-steel symmetrical cells with (a) GSE-P and (b) GCSE-P/MCN-M at various temperatures from 303 to 343 K.
